# The feasibility of utilizing the open dynamic interaction network (ODIN) app to assess rEMA data across 30 days among those recovering from alcohol use disorders

**DOI:** 10.1016/j.dadr.2024.100305

**Published:** 2024-12-04

**Authors:** Dennis E. McChargue, Bilal Khan, Jessica Phelps, Patrick Duryea, Kimberly A. Tyler, Arthur Andrews, Ellie Reznicek, Lucy Napper, Mohamed Saad, Hsuan-Wei Lee

**Affiliations:** a Department of Psychology, University of Nebraska-Lincoln, Lincoln, NE, USA; b Department of Community and Population Health & College of Computer Science and Engineering in RCEAS, Lehigh University, Bethlehem, PA, USA; c Department of Sociology, University of Nebraska-Lincoln, Lincoln, NE, USA; d Department of Psychology, Lehigh University, Bethlehem, PA, USA; e Health Data Warehouse, Lehigh University, Bethlehem, PA, USA; f Department of Population Health, Lehigh University, Bethlehem, PA, USA

**Keywords:** EMA, Alcohol use disorder, ODIN, Feasibility, Return to use, Sobriety

## Abstract

Preliminary data from a prospective micro-longitudinal study (30 days) that examined the co-evolution of return to use risk among people diagnosed with an alcohol use disorder (AUD) in residential substance treatment is presented. Data assessed the feasibility of using the open dynamic interaction network (ODIN) responsive ecological momentary assessment (rEMA). rEMA collected daily estimates on affect, urges, sober-support engagement, and use. The ODIN app administered twelve daily questions at established EMA times. GPS-identified sober support engagement and alcohol access exposure prompted additional questions. Of the eight hundred questions, most people answered 500 questions. Five-day estimates showed that 80 % of the participants answered between 80 and 100 questions (10–30 questions/day). The ODIN app acquired 95 % of GPS readings across 30 days (~288 GPS readings/day). Most were satisfied with the stability (84 %), look/feel (82 %), and ease of use (92 %) of the ODIN app. Participants also reported interest in longer assessments that prompted them to call a sponsor (85 %) or to use prevention skills (72 %). Preliminary findings show that the ODIN platform accurately and efficiently collects data amongst this population.

## Highlights

• ODIN acquired over 95 % of GPS (General Plasma Science) readings.
• 90 % of individuals answered 361–695 questions across 30 days.
• Participants were highly satisfied with ODIN app ease, stability, and look/feel (82.4–92 %).
• High percentages were interested in having the app prompt them to call sponsor (85 %) or to practice return to use prevention skills (72 %) in future studies.

## 1 Introduction

Standard practice across many countries is to discharge individuals with AUD post-detoxification to residential substance treatment facilities (Horák and Verter, 2022, Jouet et al., 2021, Mutschler et al., 2022, Santos-de-Pascual et al., 2022). Meta-analytic data shows that shorter lengths of stay during residential treatment significantly predicts return to use (Beaulieu et al., 2021). Common barriers to staying long enough include but are not limited to psychiatric (Syan et al., 2020), emotional instability (Medenblik et al., 2024, Tisdale et al., 2023), unstable living arrangements (Baker et al., 2020), inadequate sober support networks (Mohamed et al., 2022), financial instability (Pagano et al., 2021) and poverty (Patel et al., 2020, Swan et al., 2021).

Current research delineates person-environment conditions that predict returning to use by incorporating mobile ecological momentary assessment (EMA) strategies (Hutton et al., 2020, Mennis et al., 2017, Shiffman, 2009, Wrzus and Neubauer, 2023) because traditional EMA (uniform periodic sampling) may not be sufficient to determine how psychosocial factors interact with different contexts to promote/prevent substance use. For example, geographic concentration of bars and liquor stores is highly associated with AUD rates, suggesting that alcohol exposure raises risk of problematic drinking (de Medeiros et al., 2024, Gruenewald et al., 2014, Pastor et al., 2022, Theall et al., 2011), while proximity to support (e.g. support group meetings, locations of supportive friends, hobby locations that provide alcohol-free social support) has yet to receive rigorous evaluation (Stahler et al., 2013, White, 2007). These contexts are generally not considered in a traditional EMA approach. Novel approaches using mobile responsive EMA (rEMA), which supports adaptive assessments that vary in time and content of questions based on context, can assist in developing predictive models that may identify risk of returning to use “in the moment” by determining how extrinsic factors influence such behavior.

Given the importance of studying location-based EMA approaches to refine our understanding of return to use risks among those with AUDs, the purpose of the current report is to detail the feasibility of utilizing smartphone administered rEMA among individuals recovering from AUDs within local community transitional residential substance treatment facilities.

## 2 Method

### 2.1 Participant eligibility and recruitment sites

Sixty-one participants (31 males and 30 females) were recruited from one of two residential treatment facilities in the city of Lincoln, Nebraska, in the United States of America (USA) from November 2021 and March 2023. Eligibility criteria were as follows: at least 19 years of age, English speaking, comfortable using a smartphone, resident of one of the two study sites, and a primary diagnosis of severe AUD. Ethnic/racial sample composition was 74 % White, 14 % Black, 8 % Indigenous, 2 % Hispanic, and 2 % Multiracial.

The two study sites were non-hospitalized abstinence-based residential substance treatment facilities. The facilities offered alcohol and other substance treatment on-site, while encouraging participation in 12-step group meetings. These facilities provide a step between in-patient, residential treatment, and independent living for individuals with severe AUDs and substance use disorders (SUDs) that were unhoused and unemployed.

### 2.2 Procedure

Study participation lasted for 30 days and included one entry interview, 30 days of rEMA using the ODIN app, and one exit interview. Entry interviews lasted up to 120 minutes and included the collection of questionnaires [i.e., demographics, trauma scale (Hooper et al., 2011), affective scales (Cohen et al., 1983, Serafini et al., 2016), 12-step engagement (Klein et al., 2011), substance-related scales (Flannery et al., 1999, Hendershot et al., 2015)] and semi-structured interviews (McLellan et al., 1992). At the end of the entry interview, participants were trained on how to use the ODIN app on a smartphone with 30-day unlimited data provided by the study. Exit interviews lasted up to 45 minutes and included certain entry interview questionnaires (i.e., substance-related, affective state, and 12-step engagement scales) with satisfaction and acceptability questions listed below. Participants were remunerated up to $150: $20 for each in-person interview, $20 for returning the phone, and up to $90 for answering rEMA questions.

#### 2.2.1 rEMA via ODIN app

During a 30-day period, the ODIN app delivered questions to the participants’ smartphone at established assessment times each day (10 am, 2 pm, 8 pm). GPS-location coding identifying risk (e.g., bar) and support environments (12-step groups) prompted 10 additional location-based questions related to craving, use, and engagement in 12-step meetings. Other recovery-based support (e.g., counseling visits) was not coded. Phone notifications alerted participants to question availability and expiration. Participants accessed questions either by clicking on the message in their notification taskbar or by opening the app’s “Home” page. Time-based questions expired 3.5 hours after queried, alcohol access questions expired one hour after the queried, and sober support meeting questions expired 2.5 hours after queried. Participants were unable to answer nor earn incentives for expired questions.

Participants could earn up to $3 per day for the 30 days of participation based on the percentage of questions asked and answered. For example, if they answered 100 % of the presented questions, they would earn 100 % of the $3 for that day. If they answered 50 % of the questions, they would receive 50 % of the $3 for that day, i.e., $1.50. A full description of the rEMA questions used in this study can be found at https://www.niaaa.nih.gov/research/data-archive-and-resources/niaaa-data-archive.

### 2.3 Feasibility evaluation plan

Feasibility was determined by the average number of questions answered, the percentage of answered questions and the percentage of accurate GPS readings. Acceptability was determined by the average of five single-item questions Likert-scale questions. Three items measured the satisfaction level of stability, quality and the look/feel of the app. Additional items measured the ease of using an android device and the ODIN app. Another item measured the comfort level of using the ODIN app around other people. Participants were also asked two 10-point Likert-scale questions on how likely they would a) use the app if it prompted you to call your sponsor in high-risk situations, and b) use the app if it prompted you to practice prevention skills during high-risk situations. Lastly, we asked two yes/no questions about the participants’ interest in future studies, that a) paid $240 for 60 days and b) paid $330 for 90 days.

## 3 Results

### 3.1 Feasibility indicators

**Fig. 1:**
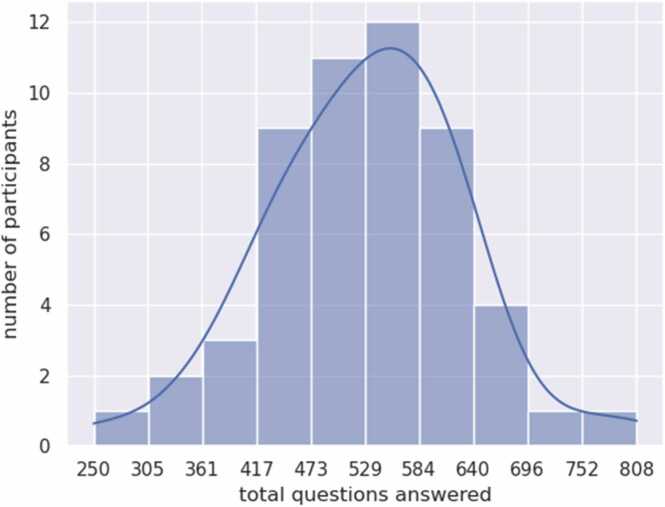
Number of questions answered across 30 days via ODIN.

On average, the ODIN app administered ~550 questions to each participant, with 90 % of individuals presented between 392 and 706 questions across 30 days of participation. In response, participants answered an average of ~550 questions each, with 90 % of individuals answering between 361 and 695 questions across 30 days of participation (Fig. 1). The mean daily response rate exhibited a modest decline, from 95 % to 85 % over 30 days of participation. The standard deviation of response rate across participants was between 5 % and 10 % every day. Overall, the distribution of participant responses showed one outlier with most answering 95 % of the questions. We observed that most individuals (94 %) maintained a response rate of ~85–100 % across the study period, though 4 individuals experienced a dip in response rates at various points in their 30 days of participation.

**Fig. 2:**
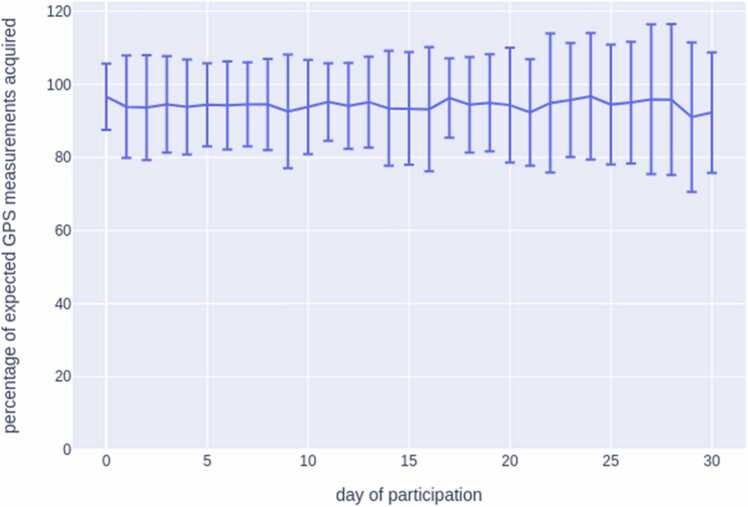
Mean percentage of expected GPS measurements acquired across 30 days. *Note:* The ODIN app takes GPS readings every five minutes. Standard deviation (error bars) across participants was ~10 % due in large part to the fact that participants were able to disable/enable GPS at any time.

The ODIN app took GPS readings every 5 minutes to evaluate for the triggering of location-based question. Fig. 2 shows that ~95 % of the expected number of daily GPS readings (12 ×24 = 288) were made, and that this percentage did not drop significantly across participants’ 30 days of participation. The standard deviation of GPS readings across participants was ~10 % because participants were able to disable/enable GPS at any time.

#### 3.1.1 Acceptability indicators

Of the 61 participants, 51 individuals completed the exit interview. No significant demographic differences existed between exit interview completers and non-completers. Acceptability data shows that most felt extremely satisfied (rating of 5 of 1–5 scale) with the a) stability (84 %; *M* = 4.37; *SD* = 0.76), b) look and feel (82 %; *M* = 4.19; *SD* = 0.55), c) ease of use (92 %; *M* = 4.12; *SD* = 0.39), d) overall quality (86 %; *M* = 4.23; *SD* = 0.81) and e) comfort level (84 %; *M* = 4.47; *SD* = 1.2). All exit interview participants (100 %) acknowledged that they would join a study that had longer assessment periods (i.e., sixty & ninety days) that also paid proportionally for participation (i.e., $240 & $330). Lastly, 85 % of participants rated eight or above on a 10-point scale that they would be interested in a future study that had the app prompt them to call their sponsor in high-risk situations. Seventy-two percent rated similarly that they would be interested in a study that had the app prompt them to use prevention skills during high-risk situations.

## 4 Discussion

The high rate of location-based responses juxtaposed with the 95 % accuracy of GPS readings and participants high acceptance towards the app shows that mobile rEMA is both feasible and well accepted. Our response rate and participant satisfaction were higher when compared with telephone-based experience sampling (Runyan et al., 2024, Swartz et al., 2023, Weiss et al., 2022) and conventional EMA sampling (Carreiro et al., 2020, Stull et al., 2023) within recovering populations. While the reasons for this are unclear, it is plausible that the notification system and ease of use promoted greater responding as compared to having to answer a phone call. Moreover, participants’ ability to have a wider answering period may have increased response rates. Nevertheless, such findings suggest that the ODIN app may offer an ideal structure to collect rEMA assessments among this population.

This study builds a platform for future use of assessment prompted just-in-time adaptive interventions (JITAI) within the rEMA framework. Consistent with JITAI development directions, our data shows that a high percentage of participants reported being interested in either having the app prompt them to call their sponsor or to practice prevention skills during high-risk situations. Furthermore, because all participants acknowledged an interest in studies involving longer assessment periods, our data implies the potential use of machine learning applications to develop such JITAIs.

### 4.1 Limitations

We tone our interpretation considering certain limitations. This study collected data from a relatively small number of individuals and the data was confined to a residential treatment population. Further, female residents were required to hand in their phones when they were staying at the treatment facility overnight. However, the influence of this limitation was minimized by the fact that no time-based questions were asked after 8 pm. Given that not all recovery support systems were able to be coded, interpretations are also limited to 12-step group attendance. Finally, there was limited racial diversity in the sample and no questions were asked to determine sexual orientation or non-binary gender.

## 5 Conclusions

This study points to the feasibility and acceptability of using the ODIN app as a tool to collect rEMA data among recovering populations. Future studies are needed to determine the ability of the ODIN app to predict drug seeking responses before they occur.

## Author disclosures

The content is solely the responsibility of the authors and does not necessarily represent the official views of the National Institutes of Health or the University of Nebraska. The authors have no relevant financial or non-financial interests to disclose. All authors contributed to the study conception and design. Material preparation, data collection and analysis were performed by Dennis McChargue, PhD, Bilal Khan, PhD, Patrick Duryea, MA, Kimberly Tyler, Arthur Andrews and Jessica Phelps, BA. The first draft of the manuscript was written by Dennis McChargue, Patrick Duryea and Jessica Phelps and authors provided significant edits to all versions of the manuscript, including Lucy Napper, Mohamed Saad and Hsuan-Wei Lee. All authors read and approved the final manuscript.

## CRediT authorship contribution statement

**Ellie Reznicek:** Writing – review & editing, Writing – original draft. **Arthur Andrews:** Writing – review & editing, Writing – original draft, Conceptualization. **Kimberly A. Tyler:** Writing – review & editing, Writing – original draft, Methodology. **Patrick Duryea:** Writing – review & editing, Writing – original draft. **Jessica Phelps:** Writing – original draft, Project administration. **Bilal Khan:** Writing – review & editing, Writing – original draft, Methodology, Investigation, Funding acquisition, Formal analysis, Data curation. **Dennis McChargue:** Writing – review & editing, Writing – original draft, Visualization, Supervision, Project administration, Methodology, Investigation, Funding acquisition, Formal analysis, Data curation, Conceptualization. **Hsuan-Wei Lee:** Formal analysis. **Mohamed Saad:** Formal analysis. **Lucy Napper:** Writing – review & editing, Writing – original draft.

## Declaration of Competing Interest

This work was supported by 10.13039/100000002NIH (R21AA029231) and the National Institute of General Medical Sciences of the National Institutes of Health [P20GM130461] and the Rural Drug Addiction Research Center at the 10.13039/100008114University of Nebraska-Lincoln. There are no financial or personal interests associated with the outcome of the study that would negatively impact the authors objectivity.
