# Supplementary material for: The feasibility of utilizing the open dynamic interaction network (ODIN) app to assess rEMA data across 30 days among those recovering from alcohol use disorders
Source: Drug Alcohol Depend Rep. 2024 Dec 4;14:100305. doi: 10.1016/j.dadr.2024.100305 (PMC11665290; doi:10.1016/j.dadr.2024.100305)
Supplement: Supplementary file 1 — Supplementary material [file mmc1.pdf]

CASA Final

LNC ODIN Platform

September 27, 2022

Contents

AA__importance **3**

access__time **4**

affect__GPS__alcohol **5**

affect__intensity **6**

affect__type **7**

alcohol__use **8**

details__GPS__support (DISABLED) **9**

details__GPS__support__updated (DISABLED) **10**

details__GPS__support__updated__08242022 (DISABLED) **11**

details__GPS__support__updated__08302022 **12**

intensity__GPS__alcohol (DISABLED) **13**

intensity__GPS__alcohol__updated__08242022 (DISABLED) **14**

intensity__GPS__alcohol__updated__08302022 **15**

stress__cause **16**

stress__extent **17**

support__details **18**

support__occurrence **19**

urge_GPS_alcohol **20**

urge_extent **21**

Study Details

Name:	CASA Final
Description:	10/29/2021 revision. Actual radii. Support locations.
Duration:	30 days

AA_importance

Column name:	AA_importance
Question Text:	On a scale from 1 to 10, where 1 is not at all important and 10 is very important, how important is it for you to attend sober support meetings?
Description:	No description provided by researcher
Question Type:	single_select

value	choice text	choice count	total percent
1	1 (not at all important)	53	6.9%
2	2	8	1.0%
3	3	12	1.6%
4	4	25	3.3%
5	5	25	3.3%
6	6	12	1.6%
7	7	33	4.3%
8	8	80	10.4%
9	9	102	13.3%
10	10 (very important)	416	54.2%
999	skipped	0	0.0%
1000	unset	1	0.1%
1001	expired	1	0.1%
	Total	768	
	Percentage		100.0%

- rule_id: 5 (Followup Rule)

Active in participant groups:	1
Time to expire:	3 hours, 30 mins
Rule-specific parameters	
Triggering question:	support_occurrence (page 19)
Delay between triggering question and this one:	0 sec(s)
Triggering answers:	1002,999

- rule_id: 6 (Followup Rule)

Active in participant groups:	1
Time to expire:	3 hours, 30 mins
Rule-specific parameters	
Triggering question:	support_details (page 18)
Delay between triggering question and this one:	0 sec(s)
Triggering answers:	any value,999

access_time

Column name:	access_time_#choice_id
Question Text:	At what times did you have access to alcohol <u>yesterday</u>, for example at a gas station or a friends house? Select all that apply.
Description:	No description provided by researcher
Question Type:	multi_select
Min:	1
Max:	6
Number of 0/1 columns:	10

choice id	choice text	choice count	total percent	fractional count	fractional percent
1	Between 12:01am and 9am	68	5.1%	38.4	4.3%
2	Between 9:01am and Noon	107	8.1%	38.0	4.2%
3	Between 12:01pm and 3pm	136	10.3%	59.2	6.6%
4	Between 3:01pm and 6pm	161	12.2%	69.5	7.8%
5	Between 6:01pm and 9pm	247	18.7%	151.5	16.9%
6	Between 9:01pm and Midnight	119	9.0%	54.5	6.1%
999	skipped	1	0.1%	1.0	0.1%
1000	unset	0	0.0%	0.0	0.0%
1001	expired	123	9.3%	123.0	13.7%
1002	I did not have access to alcohol yesterday	361	27.3%	361.0	40.3%
	Total	1323		896.0	
	Percentage				100.0%

- rule_id: 1 (Time Rule)

  Active in participant groups: 1

  Time to expire: 3 hours, 30 mins

  Rule-specific parameters

  Time schedule: At 10:00 AM

affect_GPS_alcohol

Column name:	affect_GPS_alcohol_#choice_id
Question Text:	Choose up to three emotions that you are feeling <u>right now</u>.
Description:	No description provided by researcher
Question Type:	multi_select
Min:	1
Max:	3
Number of 0/1 columns:	13

choice id	choice text	choice count	total percent	fractional count	fractional percent
1	Upset	59	3.9%	29.2	4.5%
2	Hostile	35	2.3%	16.7	2.6%
3	Alert	253	16.6%	101.8	15.8%
4	Ashamed	42	2.8%	21.0	3.3%
5	Inspired	179	11.7%	72.2	11.2%
6	Nervous	73	4.8%	31.5	4.9%
7	Determined	316	20.7%	124.5	19.3%
8	Attentive	193	12.6%	84.2	13.1%
9	Afraid	47	3.1%	19.3	3.0%
10	Active	322	21.1%	135.7	21.1%
999	skipped	0	0.0%	0.0	0.0%
1000	unset	0	0.0%	0.0	0.0%
1001	expired	8	0.5%	8.0	1.2%
	Total	1527		644.0	
	Percentage				100.0%

- rule_id: 14 (Followup Rule)

Active in participant groups:	1
Time to expire:	1 hour
Rule-specific parameters	
Triggering question:	intensity_GPS_alcohol (page 13)
Delay between triggering question and this one:	0 sec(s)
Triggering answers:	any value,999

affect_intensity

Column name:	affect_intensity
Question Text:	How are you feeling <u>right now</u>?
Description:	No description provided by researcher
Question Type:	single_select

value	choice text	choice count	total percent
1	Very good	1158	49.7%
2	Good	623	26.7%
3	Neutral	454	19.5%
4	Bad	71	3.0%
5	Very bad	17	0.7%
999	skipped	0	0.0%
1000	unset	1	0.0%
1001	expired	7	0.3%
	Total	2331	
	Percentage		100.0%

- rule_id: 9 (Followup Rule)

Active in participant groups:	1
Time to expire:	3 hours, 30 mins
Rule-specific parameters	
Triggering question:	urge_extent (page 21)
Delay between triggering question and this one:	0 sec(s)
Triggering answers:	any value,999

affect__type

Column name: affect__type__#choice__id
Question Text: Choose up to three emotions that you are feeling <u>right now</u>.
Description: No description provided by researcher
Question Type: multi__select
Min: 1
Max: 3
Number of 0/1 columns: 13

choice id	choice text	choice count	total percent	fractional count	fractional percent
1	Upset	242	4.5%	123.0	5.3%
2	Hostile	99	1.9%	45.5	2.0%
3	Alert	797	14.9%	342.3	14.8%
4	Ashamed	130	2.4%	59.2	2.6%
5	Inspired	742	13.9%	323.0	13.9%
6	Nervous	313	5.9%	143.0	6.2%
7	Determined	1000	18.7%	426.5	18.4%
8	Attentive	840	15.7%	356.0	15.4%
9	Afraid	145	2.7%	60.8	2.6%
10	Active	1029	19.3%	433.7	18.7%
999	skipped	2	0.0%	2.0	0.1%
1000	unset	0	0.0%	0.0	0.0%
1001	expired	3	0.1%	3.0	0.1%
	Total	5342		2318.0	
	Percentage				100.0%

- rule_id: 10 (Followup Rule)

Active in participant groups: 1
Time to expire: 3 hours, 30 mins

Rule-specific parameters
Triggering question: affect__intensity (page 6)
Delay between triggering question and this one: 0 sec(s)
Triggering answers: any value,999

alcohol_use

Column name:	alcohol_use
Question Text:	How many alcoholic drinks did you have <u>yesterday</u>?
Description:	No description provided by researcher
Question Type:	single_select

value	choice text	choice count	total percent
1	1 drink or less than a full drink	6	0.8%
2	2-3 drinks	4	0.5%
3	4-5 drinks	7	0.9%
4	6 or more drinks	4	0.5%
999	skipped	0	0.0%
1000	unset	0	0.0%
1001	expired	5	0.6%
1002	I did not drink alcohol yesterday	747	96.6%
	Total	773	
	Percentage		100.0%

- rule_id: 2 (Followup Rule)

Active in participant groups:	1
Time to expire:	3 hours, 30 mins
Rule-specific parameters	
Triggering question:	access_time (page 4)
Delay between triggering question and this one:	0 sec(s)
Triggering answers:	any value,999

details_GPS_support (DISABLED)

Column name: details_GPS_support_#choice_id
Question Text: If you attended a sober support meeting in the <u>last three hours</u>, please indicate which of the following activities you were involved in? Select all that apply.
Description: No description provided by researcher
Question Type: multi_select
Min: 1
Max: 5
Number of 0/1 columns: 9

choice id	choice text	choice count	total percent	fractional count	fractional percent
1	Mostly listened to others	5	55.6%	3.8	63.9%
2	Talked during breaks	1	11.1%	0.3	5.6%
3	Obtained or talked with a sponsor	0	0.0%	0.0	0.0%
4	Spoke at the meeting	2	22.2%	0.8	13.9%
5	Took a service position	0	0.0%	0.0	0.0%
999	skipped	0	0.0%	0.0	0.0%
1000	unset	0	0.0%	0.0	0.0%
1001	expired	1	11.1%	1.0	16.7%
1002	I did not attend any meeting	0	0.0%	0.0	0.0%
	Total	9		6.0	
	Percentage				100.0%

details_GPS_support_updated (DISABLED)

Column name:	details_GPS_support_updated_#choice_id
Question Text:	If you attended a sober support meeting in the <u>last three hours</u>, please indicate which of the following activities you were involved in? Select all that apply.
Description:	No description provided by researcher
Question Type:	multi_select
Min:	1
Max:	5
Number of 0/1 columns:	9

choice id	choice text	choice count	total percent	fractional count	fractional percent
1	Mostly listened to others	44	63.8%	40.8	69.1%
2	Talked during breaks	10	14.5%	5.8	9.7%
3	Obtained or talked with a sponsor	1	1.4%	0.2	0.4%
4	Spoke at the meeting	11	15.9%	9.2	15.7%
5	Took a service position	0	0.0%	0.0	0.0%
999	skipped	0	0.0%	0.0	0.0%
1000	unset	0	0.0%	0.0	0.0%
1001	expired	3	4.3%	3.0	5.1%
1002	I did not attend any meeting	0	0.0%	0.0	0.0%
	Total	69		59.0	
	Percentage				100.0%

details_GPS_support_updated_08242022 (DISABLED)

Column name: details_GPS_support_updated_08242022_#choice_id
Question Text: If you attended a sober support meeting in the <u>last three hours</u>, please indicate which of the following activities you were involved in? Select all that apply.
Description: No description provided by researcher
Question Type: multi_select
Min: 1
Max: 5
Number of 0/1 columns: 9

choice id	choice text	choice count	total percent	fractional count	fractional percent
1	Mostly listened to others	0	0.0%	0.0	0.0%
2	Talked during breaks	0	0.0%	0.0	0.0%
3	Obtained or talked with a sponsor	0	0.0%	0.0	0.0%
4	Spoke at the meeting	0	0.0%	0.0	0.0%
5	Took a service position	0	0.0%	0.0	0.0%
999	skipped	0	0.0%	0.0	0.0%
1000	unset	0	0.0%	0.0	0.0%
1001	expired	0	0.0%	0.0	0.0%
1002	I did not attend any meeting	0	0.0%	0.0	0.0%
	Total	0		0.0	
	Percentage				0.0%

details__GPS__support__updated__08302022

Column name:	details__GPS__support__updated__08302022__#choice__id
Question Text:	If you attended a sober support meeting in the <u>last three hours</u>, please indicate which of the following activities you were involved in? Select all that apply.
Description:	No description provided by researcher
Question Type:	multi__select
Min:	1
Max:	5
Number of 0/1 columns:	9

choice id	choice text	choice count	total percent	fractional count	fractional percent
1	Mostly listened to others	5	35.7%	4.5	37.5%
2	Talked during breaks	3	21.4%	2.0	16.7%
3	Obtained or talked with a sponsor	0	0.0%	0.0	0.0%
4	Spoke at the meeting	4	28.6%	3.5	29.2%
5	Took a service position	0	0.0%	0.0	0.0%
999	skipped	0	0.0%	0.0	0.0%
1000	unset	0	0.0%	0.0	0.0%
1001	expired	2	14.3%	2.0	16.7%
1002	I did not attend any meeting	0	0.0%	0.0	0.0%
	Total	14		12.0	
	Percentage				100.0%

- rule__id: 39 (GPS Rule)

  Active in participant groups: 1
  Time to expire: 2 hours, 30 mins

  Rule-specific parameters
  Locations: [{"lat":40.14349646,"long":-97 ... 40,"sched":{"0 19 * * 5":60}}]
  text-truncated (length: 6909)

intensity_GPS_alcohol (DISABLED)

Column name:	intensity_GPS_alcohol
Question Text:	How are you feeling <u>right now</u>?
Description:	No description provided by researcher
Question Type:	single_select

value	choice text	choice count	total percent
1	Very good	464	53.3%
2	Good	119	13.7%
3	Neutral	55	6.3%
4	Bad	14	1.6%
5	Very bad	6	0.7%
999	skipped	1	0.1%
1000	unset	6	0.7%
1001	expired	206	23.7%
	Total	871	
	Percentage		100.0%

intensity__GPS__alcohol__updated__08242022 (DISABLED)

Column name:	intensity__GPS__alcohol__updated__08242022
Question Text:	How are you feeling <u>right now</u>?
Description:	No description provided by researcher
Question Type:	single__select

value	choice text	choice count	total percent
1	Very good	0	0.0%
2	Good	0	0.0%
3	Neutral	0	0.0%
4	Bad	0	0.0%
5	Very bad	0	0.0%
999	skipped	0	0.0%
1000	unset	0	0.0%
1001	expired	0	0.0%
	Total	0	
	Percentage		0.0%

intensity__GPS__alcohol__updated__08302022

Column name:	intensity__GPS__alcohol__updated__08302022
Question Text:	How are you feeling <u>right now</u>?
Description:	No description provided by researcher
Question Type:	single__select

value	choice text	choice count	total percent
1	Very good	3	14.3%
2	Good	0	0.0%
3	Neutral	4	19.0%
4	Bad	1	4.8%
5	Very bad	0	0.0%
999	skipped	0	0.0%
1000	unset	0	0.0%
1001	expired	13	61.9%
	Total	21	
	Percentage		100.0%

- rule__id: 40 (GPS Rule)

Active in participant groups:	1
Time to expire:	1 hour
Rule-specific parameters	
Locations:	[{"lat":40.73766217,"long":-96 ... 0,"sched":{"0 11 * * *":480}}] text-truncated (length: 38222)

stress__cause

Column name:	stress__cause__#choice__id
Question Text:	Choose up to three things that are stressing you out <u>right now</u>.
Description:	No description provided by researcher
Question Type:	multi__select
Min:	1
Max:	3
Number of 0/1 columns:	14

choice id	choice text	choice count	total percent	fractional count	fractional percent
1	Family relationships	599	12.4%	265.7	11.6%
2	Financial	998	20.6%	365.2	16.0%
3	Work	986	20.4%	412.8	18.1%
4	Transportation	838	17.3%	373.7	16.3%
5	Alcohol use treatment	94	1.9%	49.0	2.1%
6	Healthcare access	68	1.4%	28.8	1.3%
7	Physical Health	163	3.4%	72.0	3.1%
8	Friends	157	3.2%	64.3	2.8%
9	Legal	452	9.3%	213.5	9.3%
10	Other	121	2.5%	77.0	3.4%
999	skipped	1	0.0%	1.0	0.0%
1000	unset	0	0.0%	0.0	0.0%
1001	expired	4	0.1%	4.0	0.2%
1002	Nothing	359	7.4%	359.0	15.7%
	Total	4840		2286.0	
	Percentage				100.0%

- rule__id: 12 (Followup Rule)

Active in participant groups:	1
Time to expire:	3 hours, 30 mins
Rule-specific parameters	
Triggering question:	stress__extent (page 17)
Delay between triggering question and this one:	0 sec(s)
Triggering answers:	any value,999

stress_extent

Column name: stress_extent
Question Text: On a scale of 1-10, where 1 is the least stressed and 10 is the most stressed, how stressed are you <u>right now</u>?
Description: No description provided by researcher
Question Type: single_select

value	choice text	choice count	total percent
1	1 (least stressed)	1000	43.5%
2	2	288	12.5%
3	3	312	13.6%
4	4	189	8.2%
5	5	191	8.3%
6	6	112	4.9%
7	7	93	4.0%
8	8	65	2.8%
9	9	19	0.8%
10	10 (most stressed)	25	1.1%
999	skipped	1	0.0%
1000	unset	0	0.0%
1001	expired	6	0.3%
	Total	2301	
	Percentage		100.0%

- rule_id: 11 (Followup Rule)

Active in participant groups: 1
Time to expire: 3 hours, 30 mins

Rule-specific parameters
Triggering question: affect_type (page 7)
Delay between triggering question and this one: 0 sec(s)
Triggering answers: any value,999

support_details

Column name: support_details_#choice_id
Question Text: What activities were you involved in during the sober support meeting(s) you attended <u>yesterday</u>? Select all that apply.
Description: No description provided by researcher
Question Type: multi_select
Min: 1
Max: 5
Number of 0/1 columns: 8

choice id	choice text	choice count	total percent	fractional count	fractional percent
1	Mostly listened to others	246	45.8%	217.7	49.7%
2	Talked during breaks	69	12.8%	45.0	10.3%
3	Obtained or talked with a sponsor	33	6.1%	22.8	5.2%
4	Spoke at the meeting	175	32.6%	142.2	32.5%
5	Took a service position	14	2.6%	10.3	2.4%
999	skipped	0	0.0%	0.0	0.0%
1000	unset	0	0.0%	0.0	0.0%
1001	expired	0	0.0%	0.0	0.0%
	Total	537		438.0	
	Percentage				100.0%

- rule_id: 4 (Followup Rule)

Active in participant groups: 1
Time to expire: 3 hours, 30 mins

Rule-specific parameters
Triggering question: support_occurrence (page 19)
Delay between triggering question and this one: 0 sec(s)
Triggering answers: 1,2,3,4,5,6

support_occurrence

Column name:	support_occurrence_#choice_id
Question Text:	At what times did you attend a sober support meeting <u>yesterday</u>? Select all that apply.
Description:	No description provided by researcher
Question Type:	multi_select
Min:	1
Max:	6
Number of 0/1 columns:	10

choice id	choice text	choice count	total percent	fractional count	fractional percent
1	Between 12:01am and 9am	12	1.4%	12.0	1.6%
2	Between 9:01am and Noon	58	6.8%	40.9	5.3%
3	Between 12:01pm and 3pm	54	6.4%	38.2	5.0%
4	Between 3:01pm and 6pm	57	6.7%	38.8	5.1%
5	Between 6:01pm and 9pm	307	36.2%	283.8	37.0%
6	Between 9:01pm and Midnight	31	3.7%	24.2	3.1%
999	skipped	1	0.1%	1.0	0.1%
1000	unset	0	0.0%	0.0	0.0%
1001	expired	0	0.0%	0.0	0.0%
1002	I did not attend a meeting yesterday	329	38.8%	329.0	42.8%
	Total	849		768.0	
	Percentage				100.0%

- rule_id: 3 (Followup Rule)

Active in participant groups:	1
Time to expire:	3 hours, 30 mins
Rule-specific parameters	
Triggering question:	alcohol_use (page 8)
Delay between triggering question and this one:	0 sec(s)
Triggering answers:	any value,999

urge_GPS_alcohol

Column name: urge_GPS_alcohol
Question Text: Please indicate how strong your urge is to drink alcohol <u>right now</u>, where 0 is no urge and 10 is the strongest urge.
Description: No description provided by researcher
Question Type: single_select

value	choice text	choice count	total percent
1	0 (no urge)	497	78.9%
2	1	28	4.4%
3	2	20	3.2%
4	3	24	3.8%
5	4	17	2.7%
6	5	28	4.4%
7	6	3	0.5%
8	7	8	1.3%
9	8	0	0.0%
10	9	0	0.0%
11	10 (strongest urge)	1	0.2%
999	skipped	1	0.2%
1000	unset	0	0.0%
1001	expired	3	0.5%
	Total	630	
	Percentage		100.0%

- rule_id: 15 (Followup Rule)

Active in participant groups: 1
Time to expire: 1 hour

Rule-specific parameters
Triggering question: affect_GPS_alcohol (page 5)
Delay between triggering question and this one: 0 sec(s)
Triggering answers: any value,999

urge_extent

Column name:	urge_extent
Question Text:	Please indicate how strong your urge is to drink alcohol <u>right now</u>, where 0 is no urge and 10 is the strongest urge.
Description:	No description provided by researcher
Question Type:	single_select

value	choice text	choice count	total percent
1	0 (no urge)	1690	66.2%
2	1	185	7.2%
3	2	146	5.7%
4	3	96	3.8%
5	4	67	2.6%
6	5	75	2.9%
7	6	26	1.0%
8	7	14	0.5%
9	8	8	0.3%
10	9	8	0.3%
11	10 (strongest urge)	22	0.9%
999	skipped	1	0.0%
1000	unset	11	0.4%
1001	expired	204	8.0%
	Total	2553	
	Percentage		100.0%

- rule_id: 7 (Time Rule)
  Active in participant groups: 1
  Time to expire: 3 hours, 30 mins

  Rule-specific parameters
  Time schedule: At 02:00 PM and 08:00 PM

- rule_id: 8 (Followup Rule)
  Active in participant groups: 1
  Time to expire: 3 hours, 30 mins

  Rule-specific parameters
  Triggering question: AA_importance (page 3)
  Delay between triggering question and this one: 0 sec(s)
  Triggering answers: any value,999

List of disabled questions

1. If you attended a sober support meeting in the <u>last three hours</u>, please indicate which of the following activities you were involved in? Select all that apply.

2. If you attended a sober support meeting in the <u>last three hours</u>, please indicate which of the following activities you were involved in? Select all that apply.

3. If you attended a sober support meeting in the <u>last three hours</u>, please indicate which of the following activities you were involved in? Select all that apply.

4. How are you feeling <u>right now</u>?

5. How are you feeling <u>right now</u>?
